# Supplementary material for: Limonium tetragonum Promotes Running Endurance in Mice through Mitochondrial Biogenesis and Oxidative Fiber Formation
Source: Nutrients. 2022 Sep 21;14(19):3904. doi: 10.3390/nu14193904 (PMC9570989; doi:10.3390/nu14193904)
Supplement: Supplementary file 1 [file nutrients-14-03904-s001.zip › nutrients-1849665-supplementary.pdf]

***Limonium tetragonum* promotes running endurance in mice through mitochondrial biogenesis and oxidative fiber formation**

Running title: *Limonium tetragonum* water extract improves endurance capacity

Contents

1. Supplementary tables
2. Supplementary figures

Supplementary tables

Table S1. Sequences of primers for qPCR

**Genes**	**Primers**	**Sequence (5'-3')**
Myh7	Forward	ACAAGCTGCAGCTGAAGGTG
	Reverse	TCATTCAGGCCCTTGGCAC
Myh2	Forward	CCAGCTGCACCTTCTCGTTTGCCAG
	Reverse	CATGGGGAAGATCTGGTCTTCTT
Tnni1	Forward	TGAAGCCAAATGCCTCCACAACAC
	Reverse	ACACCTTGTGCTTAGAGCCCAGTA
Tnnc1	Forward	AGCTCATGAAGGACGGTGACAAGA
	Reverse	AACCGTGCAAGACCAGCATCTACT
Tnnt1	Forward	AAGGGGAGCGTGTGGATTTTG
	Reverse	TCCTCCTTTTTCCGCTGTTCA
Myh4	Forward	CCTGGAACAGACAGAGAGGAGCAGGAGAG
	Reverse	GTGAGTTCCTTCACTCTGCGCTCGTGC
Myh1	Forward	GCAACAGTTCTTCAACCAC
	Reverse	GCCAGGTCCATCCCAAAGT
Tnnt3	Forward	AACTGGAGACTGACAAATTCGAGT
	Reverse	GCTGTGCTTCTGGGTTTGGT
Tnnc2	Forward	CCATCATCGAGGAGGTGGAC
	Reverse	CTTCCCCTTCGCATCCTCTT
Tnni2	Forward	GCACCTGAAGAGTGTGATGCT
	Reverse	TCTCCTTCTCAGATTCTCGGC
Ppia	Forward	GCATACGGGTCCTGGCATCTTGTCC
	Reverse	ATGGTGATCTTCTTGCTGGTCTTGC
Mb	Forward	CATGGTTGCACCGTGCTCACAG
	Reverse	GAGCCCATGGCTCAGCCCTG
Sdhb	Forward	CAGAGTCGGCCTGCAGTTTC
	Reverse	GGTCCCATCGGTAAATGGCA
Fndc5	Forward	TCCTCTTCATGTGGGCAGGT
	Reverse	GGGCTCGTTGTCCTTGATGATA
Nrf1	Forward	GGAGCACTTACTGGAGTCC
	Reverse	CTGTCCGATATCCTGGTGGT
Tfam	Forward	GCAAAGGATGATTCGGCTCAGGGAA
	Reverse	CCGGATCGTTTCACACTTCGACGG
Mtco1	Forward	CTACTATTCGGAGCCTGAGC
	Reverse	GCATGGGCAGTTACGATAAC
Mtco2	Forward	AACCATAGGGCACCAATGATAC
	Reverse	GGATGGCATCAGTTTTAAGTCC
Mcad	Forward	GGTTTGGCTTTTGGACAATG
	Reverse	TGACGTGTCCAATCTACCACA

Nfatc1	Forward	GGGACGCCCATGCAATCTGT
	Reverse	AAAATCGCAGGCTTCCCCCG
Mef2	Forward	CAACGACAGAGCCAGACAT
	Reverse	TGCCAGACGCACATCC
Ppargc1a	Forward	ACGAGGCCAGTCCTTCCTCC
	Reverse	AGCTCTGAGCAGGGACGTCT
Nr4a3	Forward	TAGCCATCCATTCTGCCTTTG
	Reverse	TGGTGTATTCCGAGCCATAAGT
Gapdh	Forward	GGCATGGACTGTGGTCATGA
	Reverse	TTCACCACCATGGAGAAGGC

Table S2. Antibody information

Antibody	Company	Catalogue Number	Dilution
MyHC1	Developmental Studies Hybridoma Bank (DSHB)	BA-D5	1:2500
MyHC2a	Developmental Studies Hybridoma Bank (DSHB)	SC-71	WB-1:1000<br>IF-1:100
MyHC2b	Developmental Studies Hybridoma Bank (DSHB)	BF-F3	WB-1:1000<br>IF-1:100
Total-MyHC	Developmental Studies Hybridoma Bank (DSHB)	MF-20-S	WB-1:1000<br>IF-1:100
Total OxPhos antibody cocktail	Abcam	ab110413	1:1000
HSP90	Enzo Life Sciences	ADI-SPA-836-F	1:2500
Mef2a	Abcam	Ab109420	1:2500
Mef2c	Abcam	Ab227085	1:2500
Mfn1	Abcam	ab567602	1:2500
OPA1	BD Biosciences	612606	1:2500
Drp1	Santa Cruz Biochemicals	sc-271583	1:2500
Fis1	Santa Cruz Biochemicals	sc-376447	1:2500
ATF2	Santa Cruz Biochemicals	sc-242	1:2500
p-ATF2	Santa Cruz Biochemicals	sc-8398	1:2500
PGC-1α	Millipore	AB-3242	1:2500
CREB	Cell Signaling Technology	9197	1:2500
p-CREB	Cell Signaling Technology	9198	1:2500
p-PKA substrates	Cell Signaling Technology	9624S	1:2500
p38MAPK	Cell Signaling Technology	9212S	1:2500
p-p38MAPK	Cell Signaling Technology	9211S	1:2500
AKT2	Cell Signaling Technology	2962	1:2500
p-AKT	Cell Signaling Technology	4060S	1:2500
LC3B	Cell Signaling Technology	2775S	1:2500
Alexa Fluor 350-conjugated goat anti-mouse IgG2b	Thermo Fisher Scientific	A21140	1:100
Alexa Fluor 488-conjugated goat anti-mouse IgG1	Thermo Fisher Scientific	A21121	1:100
Alexa Fluor 594-conjugated goat anti-mouse IgM	Thermo Fisher Scientific	A21044	1:100

Supplementary Figures

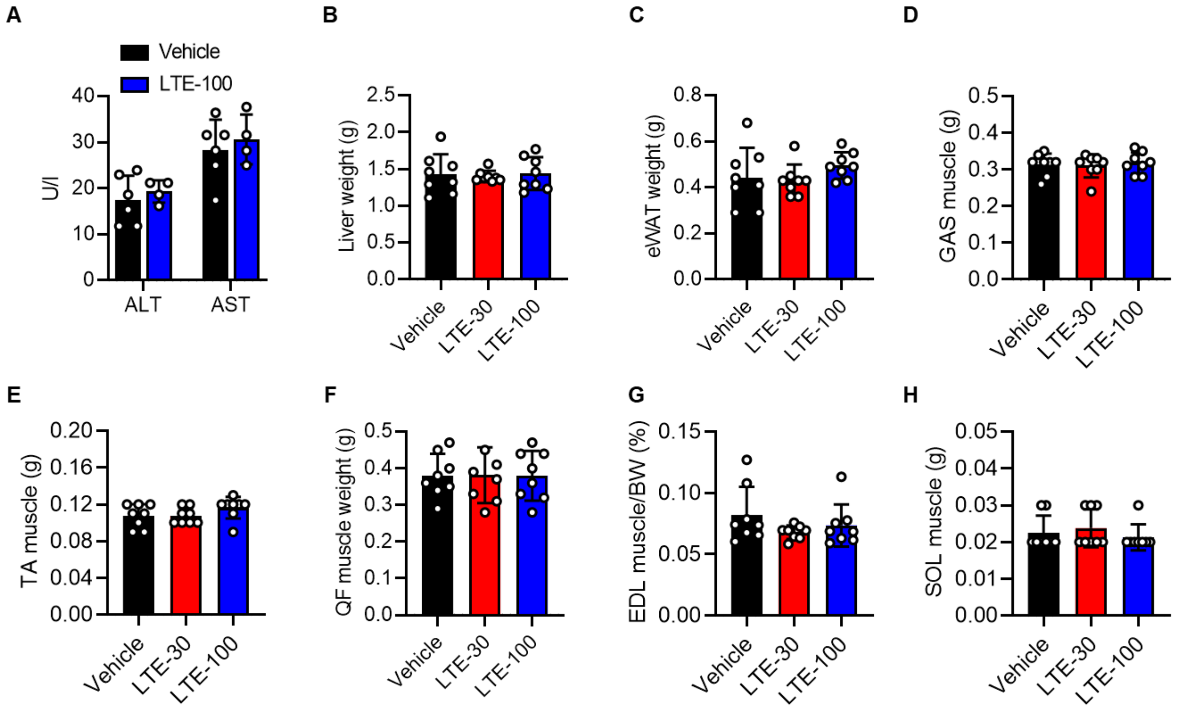

Supplementary Figure S1. No effects of four-week LTE supplementation on liver enzymes and muscle weight. (A) Liver enzymes (n = 4-6), **(B-E)** The weight of liver, epididymal white adipose tissue (eWAT) and specific muscles were compared between control and LTE-supplemented mice. Values are mean ± S.E.M. ALT, alanine aminotransferase; AST, aspartate aminotransferase; GAS, gastrocnemius (GAS); TA, tibialis anterior; QF, quadriceps femoris; EDL, extensor digitorum longus; SOL, soleus; LTE-30, LTE 30 mg/kg p.o.; LTE-100, LTE 100 mg/kg p.o.

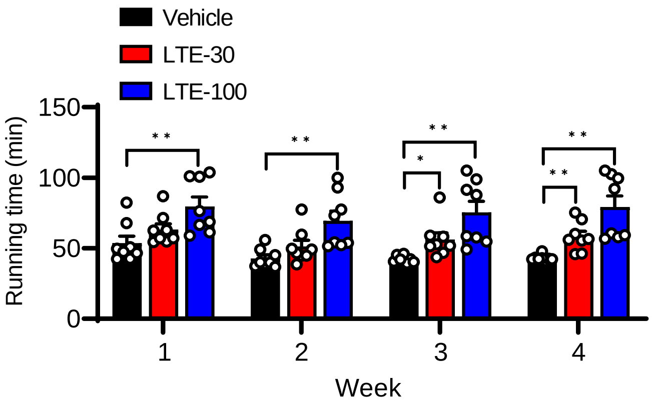

Supplementary Figure S2. Effects of LTE supplementation on treadmill running endurance. All experimental procedures were identical to those described in Figure 2 legend. Weekly average running time was measured (n = 8). Values are mean ± S.E.M., * p<0.05 and ** p<0.01. LTE-30, LTE 30 mg/kg p.o.; LTE-100, LTE 100 mg/kg p.o.

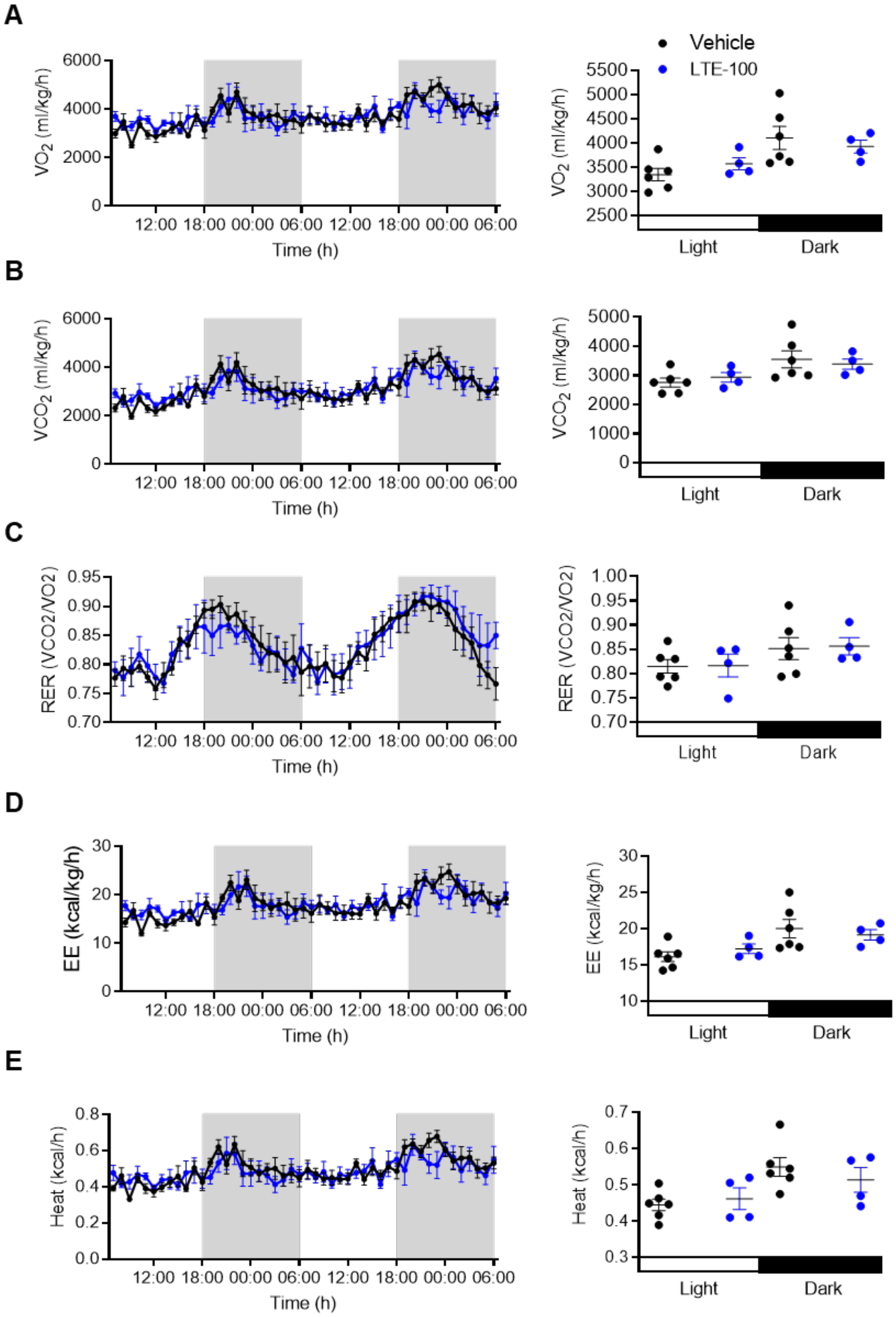

Supplementary Figure S3. Indirect calorimetry analysis. Indirect calorimetry was performed using an 8-chamber Oxymax system. Mice were acclimatized to cages for 24 h and data was collected for an additional 24 h. Twenty-four hour oxygen consumption rates (VO_2, **A**), carbon dioxide production rates (VCO_2, **B**), respiratory exchange ratio (RER, **C**), average energy expenditure (EE, **D**) and heat production in mice (n=4-6). Values are mean ±SD.

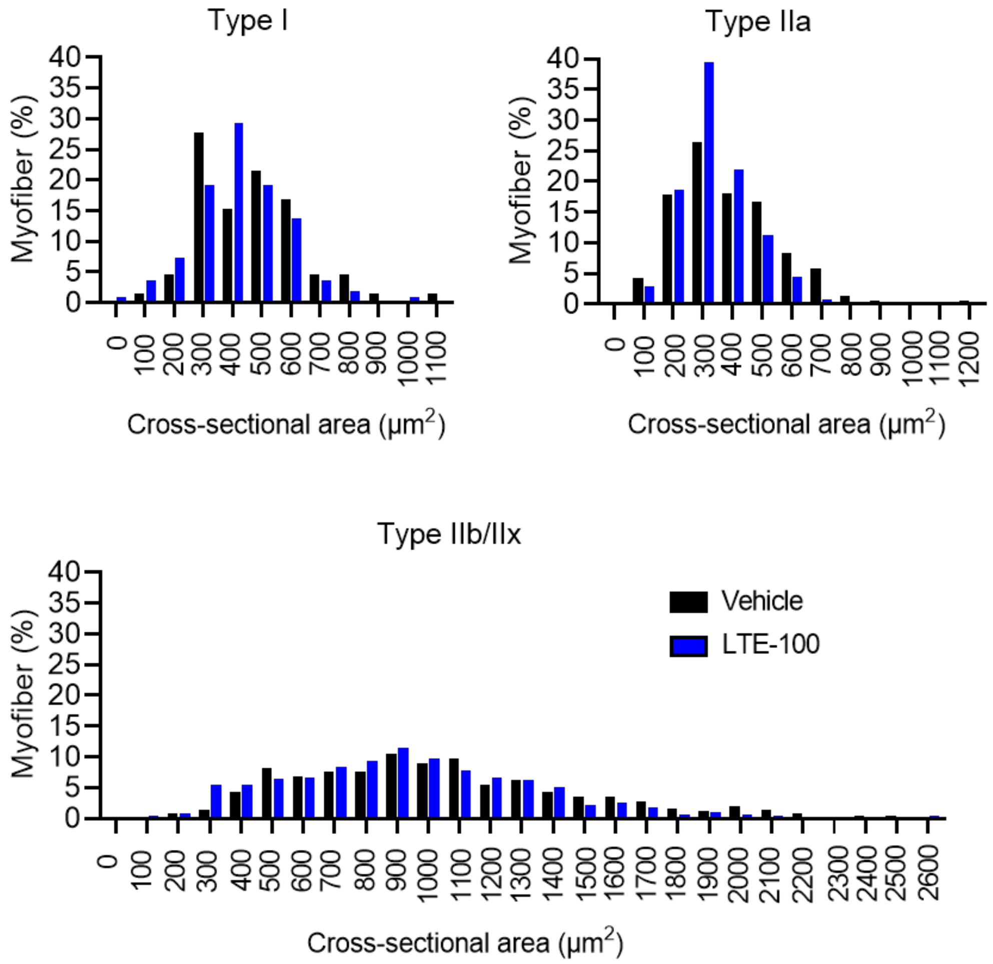

Supplementary Figure S4. Myofiber size distribution histograms in GAS muscles of control and LTE-supplemented mice. The cross-sectional area of each type of muscle fiber was determined based on the expression of MyHC-positive myofibers in Figure 3A.

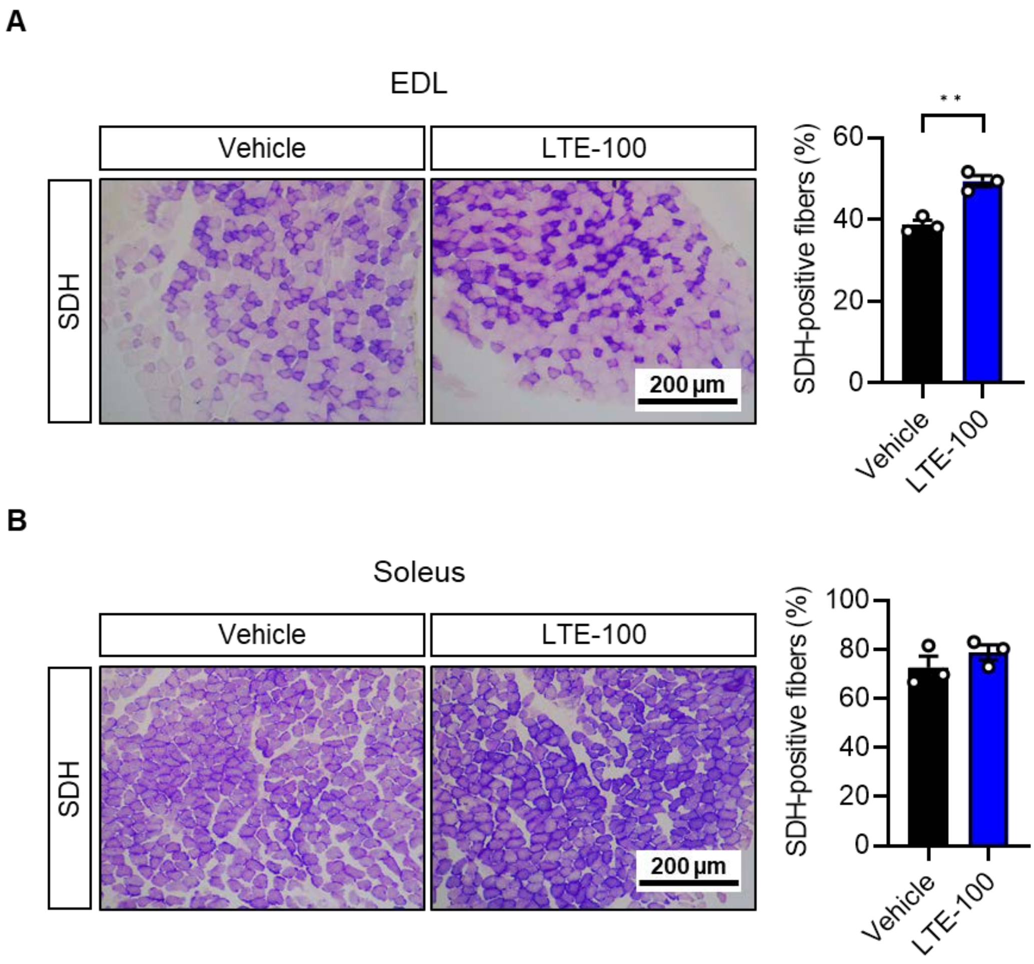

Supplementary Figure S5. Increase in oxidative fiber density in the extensor digitorum longus (EDL) muscle of LTE-supplemented mice. All experimental procedures were identical to those described in Figure 2 legend. Succinate dehydrogenase (SDH) staining and quantification of SDH-positive fibers (n=3). Values are mean ± SEM. **, $p<0.01$.

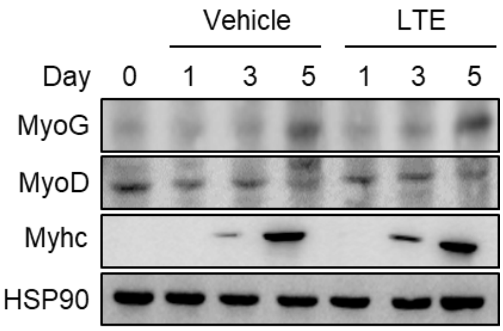

Supplementary Figure S6. No effects of LTE on myogenic differentiation. C2C12 myoblasts were incubated with LTE 30 μg/ml for 5 days in the differentiation medium. Protein levels of myogenic markers were determined by Western blotting.

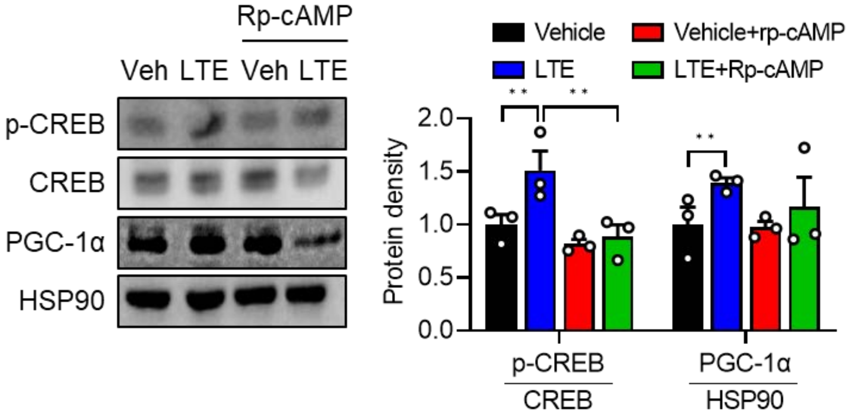

Supplementary Figure S7. Effects of PKA inhibitor Rp-cAMP on CREB-PGC-1α axis. C2C12 myoblasts were incubated with LTE 30 μg/ml with or without Rp-cAMP (300 μM) for 5 days in the differentiation medium. Protein levels of CREB and PGC-1α were determined by Western blotting.
